# Evidence synthesis papers would benefit from segmented peer review

**DOI:** 10.1016/j.tranon.2021.101060

**Published:** 2021-03-08

**Authors:** Kate Nyhan, Holly K. Grossetta Nardini

**Affiliations:** 1 Harvey Cushing/John Hay Whitney Medical Library, Yale University; 2 Department of Environmental Health Sciences, Yale School of Public Health, Yale University

## Abstract

• Evidence synthesis (including systematic reviews, scoping reviews, and more) involves many complementary areas of expertise: methods, information retrieval, statistics, and clinical knowledge.
• Few individual peer reviewers have all the requisite knowledge to evaluate all aspects of evidence synthesis manuscripts.
• Segmented peer review as proposed by Dinakaran et al offers a good solution to the challenges of rigorously peer reviewing evidence synthesis manuscripts.

To the editor:

We read with interest the proposal for segmented peer review by Dr Dinakaran et al. [1] We agree that such a peer review process would offer real benefits. From our perspective as medical librarians with expertise in evidence synthesis methods, we have observed that many published systematic reviews pass through the peer review process at well-regarded journals even though the methods (in particular, the search strategies) are poorly designed, poorly reported, or both. Many peer reviewers and journal editors are unaware of the best practices in this aspect of evidence synthesis and thus allow low-quality research to enter the peer-reviewed literature. The authors mention multidisciplinary research as a genre that would benefit from segmented peer review, and the team science nature of systematic reviews – including domain experts, experts in methods and reporting, information retrieval specialists, and statisticians – means that systematic reviews would certainly benefit from segmented review as well.

Librarians are, of course, well positioned to evaluate complicated search strategies and the methodologies of evidence syntheses. In 2018, we surveyed medical librarians to understand their experiences as peer reviewers of systematic reviews and other evidence synthesis papers and learned that some librarians decline to review papers because they do not feel competent to evaluate the whole papers. One quote that we included in the article: “I was asked to review the entire systematic review, which I did not feel competent to do. Had they asked for the search methods/search strategy only, I would have been happy to do so.” We also offer the parallel example of statisticians, who are frequently asked to peer review sections of papers that involved complex statistics, without needing to comment on the entire manuscript. [2]

Journal editors seeking peer reviewers with expertise in evaluating systematic searches are invited to use the Librarian Peer Reviewer Database. [3] Over 150 independent reviewers can be contacted by editors with requests to peer review evidence synthesis and metaresearch manuscripts in which literature searches play an important role. Their expertise can be valuable in peer review of whole manuscripts or Dinakaran et al's proposed segmented peer review.

Segmented peer review as proposed by Dinakaran et al could reduce the burden on individual reviewers while also welcoming into the peer review ecosystem reviewers whose deep (if narrow) expertise is important in ensuring the high quality of accepted manuscripts.

## CRediT authorship contribution statement

**Kate Nyhan:** Writing – original draft, Writing – review & editing. **Holly K. Grossetta Nardini:** Writing – original draft, Writing – review & editing.
